# Low Percent Copper Doping Limits Bacterial Adhesion on Fluorapatite-Based Bone Scaffolds

**DOI:** 10.1002/jbm.b.70094

**Published:** 2026-05

**Authors:** Pooya Elahi, Samantha K. Steyl, Alec Griffin, James Peter Beck, Jay Agarwal, Jill Shea, Sujee Jeyapalina

**Affiliations:** 1 Orthopaedic and Plastic Surgery Research Laboratory, George E. Wahlen Department of Veterans Affairs Medical Center, Salt Lake City, Utah, USA; 2 Division of Plastic Surgery, Department of Surgery, University of Utah School of Medicine, Salt Lake City, Utah, USA; 3 Department of Biomedical Engineering, University of Utah, Salt Lake City, Utah, USA; 4 Department of Veteran Affairs Salt Lake City Dental Clinic, Salt Lake City, Utah, USA; 5 University of Utah School of Dentistry, Salt Lake City, Utah, USA; 6 Department of Orthopaedics, University of Utah, Salt Lake City, Utah, USA

**Keywords:** bacteriostatic surfaces, bone scaffold, cell viability, Cu-doped fluorapatite, rat tibial defect model

## Abstract

Natural and engineered bone grafts are widely used in orthopedic and dental reconstruction; however, infection remains a persistent clinical challenge. Fluorapatite (FAp) is a chemically stable and osteoconductive bone substitute, but it lacks inherent antimicrobial properties. Copper (Cu), known for its potent antibacterial activity, could be incorporated into the FAp crystal structure, yet studies on Cu-doped fluorapatite (CuFAp) are limited. Notably, our previous research demonstrated that Cu doping at 1–5 mol% concentrations generated surfaces that inhibited bacterial adhesion, but were also cytotoxic to osteoblasts, suggesting that even lower concentrations could promote osteoblast activity while preventing bacterial adhesion. Therefore, we hypothesized that doping FAp with low Cu concentrations (0.25, 0.50, 0.75, and 1.0 mol%) would result in a significant reduction in bacterial adhesion while preserving osteogenic properties. Material characterization analyses confirmed successful Cu substitution at these levels within the apatite lattice without the formation of secondary oxide phases. Surface analysis of sintered CuFAp surfaces revealed an increase in grain sizes with increasing Cu contents. Antibacterial assays using *Staphylococcus aureus* and *Pseudomonas aeruginosa* demonstrated that 0.25–0.50 mol% CuFAp reduced bacterial adhesion on their surface by up to 3 log-fold compared to undoped FAp. Crucially, osteoblast viability remained unaffected over 72 h (*p* > 0.05). Based on these results, 0.50 mol% CuFAp scaffolds were evaluated in a pilot rat model of contaminated critical-size bone defects. Histological assessment confirmed complete bone regeneration with minimal inflammation, highlighting both osteogenic and antibacterial adhesion properties of these surfaces. Collectively, these findings indicate that low-concentration CuFAp scaffolds represent a promising biomaterial for infection-resistant bone defect repair.

## 1 | Introduction

Bone grafts are frequently utilized in various orthopedic and dental procedures, with over 2 million surgeries requiring their use annually worldwide, including over 500,000 in the United States [1–5]. While autografts are considered the gold standard for bone defect repair due to their superior osteogenic potential and ability to integrate seamlessly with host tissue without eliciting an immune response [6–8], their use is limited by donor site morbidity, postoperative pain, cost, limited harvesting sites and volumes, and infection risk [6, 9–11]. These drawbacks have prompted the exploration of alternatives, including allografts, xenografts, and synthetic bone graft substitutes [7, 12].

Among synthetic graft materials, calcium phosphate-based bioceramics, such as hydroxyapatite (HAp)—which stoichiometrically mimics the inorganic component of bone—beta-tricalcium phosphate (β-TCP), and fluorapatite (FAp)—primarily found in shark tooth enamel—have garnered significant attention due to their osteoconductive, bioactive, and biocompatible properties [13, 14]. In addition, FAp is reported to have anabolic potentials while inhibiting osteoclast activities [15–22]. Moreover, FAp also offers enhanced hardness, superior resistance to acid dissolution, and higher crystallinity compared to HAp [23, 24], which contribute to its observed resistance to in vivo degradation properties [25, 26]. Collectively, these properties allow tooth enamel to withstand a lifetime of wear in a bacterially rich oral microenvironment. Literature also suggests that FAp could be equal to or superior to autografts, where maintaining the structural integrity of the scaffold during healing is critical for success [27–29]. Furthermore, multiple reports have demonstrated that fluoride ions (F^−^) stimulate osteoblast proliferation and differentiation, as well as promote bone deposition in patients with osteoporosis [30, 31]. Our previous study showed that adipose-derived stem cells, when grown on FAp scaffolds, expressed statistically improved expressions of osteogenic markers and at earlier time points than those seeded onto HAp [32]. Taken together, these properties make FAp an attractive candidate for further development as a bone replacement material.

In many applications, grafting materials are required for use in bacterially compromised sites, i.e., in dental procedures or serious orthopedic injuries, including those from accidents or massively contaminated battlefield wounds. Graft failures are often attributed to infection, poor blood supply, and graft resorption [33]. Infection-related graft failures vary widely, with rates reaching up to 25% in some cases [34]. Thus, incorporation of antimicrobial properties into FAp scaffolds could help to reduce the reported high graft failure rates due to infection, providing an additional benefit [35, 36]. Developing effective antimicrobial FAp scaffolds would therefore address a critical clinical need, motivating the current study.

Although it has been reported that FAp possesses limited antibacterial properties, these effects may be insufficient to protect grafts in minimally contaminated environments [37, 38]. Previous studies reported that the bacteriostatic property of FAp-based bone grafts can be enhanced further by low percentage antimicrobial metal doping [39, 40], through their ability to kill bacteria via ion release, reactive oxygen species generation, and interference with bacterial metabolism [41–45]. Previous research has also shown that apatite doped with < 5% molar Cu reduced bacterial adhesion of both *S. aureus* and *E. coli* [40]. It is worth noting that while FAp has been investigated individually as a potential bone-replacement scaffold [46–48] and Cu as an antimicrobial additive [49–55], research on CuFAp materials (i.e., Cu incorporated as part of the apatite crystal structure) is limited [39, 56, 57]. Also, in bone regeneration, Cu is known to play a critical role in collagen cross-linking and elastin formation, promoting structural integrity and resilience in connective tissues [3, 58–61]. Some studies have reported that Cu loss in bone is associated with marked reductions in bone density and mechanical strength, highlighting the benefits of Cu for bone health [62, 63]. These data collectively suggest that the Cu-doped FAp has the potential to exhibit antimicrobial properties while promoting skeletal tissue regeneration [64]. In our previous study, 1–5 molar percent doped CuFAps significantly reduced bacterial adhesion; however, these concentrations also exhibited cytotoxic effects on osteoblasts, impairing their differentiation and cellular functions [65]. These findings highlight the necessity of optimizing doping levels to achieve a balance between antimicrobial efficacy and host cell compatibility, thereby providing the rationale for the present research.

In this study, we hypothesized that introducing even lower concentrations of Cu dopants (0.25, 0.50, 0.75, and 1 mol%) into the FAp lattice during synthesis would limit bacterial adhesion to surfaces while promoting osteogenic cell adhesion, differentiation, and bone matrix deposition. This hypothesis was tested using planktonic bacterial cultures, osteoblastic adhesion and proliferation assays, and a pilot animal study to assess the ability of the CuFAp scaffold to regenerate bone tissue in a contaminated bone defect.

## 2 | Materials and Methods

### Chemicals:

Unless otherwise specified, all reagent-grade chemicals were purchased from Sigma-Aldrich (St. Louis, MO) for in-house apatite synthesis.

### 2.1 | Materials Synthesis and Characterization

#### Synthesis:

As previously described, FAp and CuFAp were synthesized using a wet chemistry-precipitation method [66]. Briefly, solutions of calcium nitrate tetrahydrate, (Ca(NO_3_)_2_·4H_2_O) and Cu nitrate trihydrate (Cu(NO_3_)_2_·3H_2_O) were prepared. Volumes of these nitrate solutions needed to achieve the desired stoichiometric composition of Ca_(10-2*x*)_Cu_2*x*_(PO_4_)_6_F_2_ (where *x* = 0.0125, 0.025, 0.0375, or 0.05, corresponding to 0.25, 0.5, 0.75, and 1 mol% CuFAp, respectively) were measured out and mixed. Additionally, a solution containing sodium phosphate dibasic (Na_2_HPO_4_) and sodium fluoride (NaF) was prepared to provide phosphate and fluoride ions needed for apatite synthesis. These solutions were added dropwise to a reaction vessel containing deionized water (DIW) at 96°C, maintained at pH 9.2, with a controlled flow rate of 2.8 mL/min. Following synthesis, the resulting powder was washed with 20 L of DIW to remove all soluble byproducts (i.e., salts).

#### Characterization:

X-ray diffraction (XRD) patterns of the as-synthesized powders were obtained using a diffractometer (Bruker D8 Discover; Billerica, MA), with X-ray generated at 40 kV and 40 mA using Ni-filtered Cu-K_α_ radiation (*λ* = 1.5418 Å; i.e., weight average of Cu-K_α1_ (1.5404 Å) and Cu-K_α2_ (1.5443 Å)) in the 2*θ* scanning range of 10° to 80°. Phase identification, quantification, and crystal parameter calculation were performed based on reference intensity ratio (RIR) values. Data smoothing and Kα_2_-stripping were applied to optimize the fit. Full-pattern Rietveld refinement was conducted using Bruker Diffrac.Eva software (Billerica, MA). Phase identification and crystallinity were determined by comparing the XRD patterns to reference data from the Crystallography Open Database (COD; entry 96–900-1881) for FAp. The average crystallite size was then calculated using the Debye–Scherrer equation (Equation 1):

(1) $$D_{c}=\frac{A\lambda }{\beta \;cos\;\theta }$$

where $D_{c}$ is the crystallite size (nm), A is the shape factor (unitless), λ is the X-ray wavelength (nm), β is the full width at half maximum (radians), and θ is the Bragg angle (radians).

The oven-dried, finely ground potassium bromide and apatite powders (300:1 ratio) were manually pelletized using vacuum dies and a hydraulic press (Specac; Fort Washington, PA) at 300 MPa. The functional groups of the thin disks were determined using Fourier-transform infrared spectroscopy (FTIR; ThermoFisher Scientific Nicolet iS50; Waltham, MA) with a signal-to-noise ratio of 50,000:1 (peak-to-peak) between wave-numbers 400–4000 cm^−1^.

The Cu, calcium, and phosphate ion concentrations in FAp and CuFAp powders were quantified using inductively coupled plasma-optical emission spectroscopy (ICP-OES) (Agilent 5800; Thermo Scientific; Waltham, MA). For this analysis, approximately 30–50 mg of CuFAp or FAp were weighed into acid-cleaned Teflon digestion vessels. The samples were digested with a concentrated HNO_3_/HF mixture at a 3:1 volume ratio. The vessels were sealed and heated in a microwave digestion system at 180°C for 30 min to fully dissolve the samples. After digestion, the solutions were evaporated to dryness using a hot plate at 150°C, then redissolved in 5 mL of 2% HNO_3_. The solutions were transferred to polypropylene tubes and diluted with ultrapure water to a final volume of 50 mL. Calibration standards were made from certified multielement stock solutions and diluted in 2% HNO_3_ to match the sample matrix. Procedural blanks were prepared and processed in the same way as the samples. Elemental concentrations were measured using an ICP–OES instrument under standard conditions. Samples were nebulized and introduced into an argon plasma, and emission intensities were measured at wave-lengths chosen to reduce spectral interferences. Agilent ICP Expert Pro software was used to control the 5800 ICP-OES instrument, optimize and run the method, and process data for the following analytes: calcium (wavelengths 317.933 and 422.673 nm), Cu (wavelength 327.395 nm), and phosphorus (wavelengths 177.434 and 213.618 nm).

The surface topographies of the samples were characterized using scanning electron microscopy (SEM) (FEIQuanta600 FEG, Hillsboro, OR). Before imaging, the pellets were fabricated as described above using a conventional die-press method [67], and sintered at 1150°C for 2 h and a ramp rate of 2°C/min. The SEM analysis was conducted on these sintered pallets after cleaning with ethanol. The average grain size was determined using the mean linear intercept method with ImageJ software (NIH-LOCI) [68].

### 2.2 | Test Samples Preparation

Disks were fabricated and sintered at 1150°C, as described previously [67]. Prior to bacterial culture studies, the disks also underwent sonication in deionized water to remove loosely adhered particles, then dehydrated using a graded ethanol series, air-dried, and steam-sterilized.

### 2.3 | Evaluation of Bacterial Adhesion Properties on Apatite Surfaces

Bone infections can result from both Gram-negative and Gram-positive bacteria, each exhibiting varying degrees of antibiotic responses and distinct mechanisms of action. Therefore, it is necessary to evaluate the adhesion capabilities of both bacterial types to CuFAp surfaces. *S. aureus* and *P. aeruginosa*, as primary agents of bone graft infections [69–71], were selected as model organisms for this study. Using standard techniques, 5 × 10^8^ CFU/mL of bacterial concentrations were made from freshly frozen isolates of *S. aureus* (ATCC 25923) and *P. aeruginosa* (ATCC 15442). CuFA disks (*n* = 4/group) were placed into an individual well of a 12-well plate. Then, 50 μL of the bacterial suspension was carefully dropped onto the top planar surface of each disk to ensure uniform bacterial coverage. The disks were incubated at 37°C for 2 h. Following incubation, the disks were gently washed with PBS to remove unattached bacteria and subsequently transferred into 1 mL of fresh PBS. Each sample underwent vortexing for 30 s, followed by 5 min of ultrasonication in an ultrasonic bath (Branson 3800, Emerson Electric Co., St. Louis, MO) at 40 kHz and a final 30 s of vortexing. A standard serial dilution procedure was then performed on the resulting suspension to quantify the number of adhered bacteria on each surface.

### 2.4 | Cell Adhesion and Differentiation Assays

To assess the cytocompatibility of sintered CuFAp and control (FAp) surfaces, the transfected human fetal osteoblast cells (passages 4–7; hFOB 1.19, CRL-11372, ATCC, Manassas, VA) were used. Four samples per group were used, and all experiments were done in triplicate. Initially, complete osteoblast growth media was prepared with phenol free F12/Dulbecco's Modified Eagle Medium (DMEM; 21041–025, Gibco, Billings, MT) that was supplemented with 10% heat-inactivated fetal bovine serum (Thermo Fisher Scientific, Waltham, MA), 25 mM L-glutamine (Gibco, Thermo Fisher Scientific), and 0.3 mg/mL Geneticin selective antibiotic (G418 disulfate salt, Sigma-Aldrich).

After recovering osteoblast cells from cryopreservation and expanding them, a suspension containing ~18 k cells/cm^2^ was prepared with complete osteoblast growth media using standard procedures. After placing 100 μL of the above cell suspension as a droplet onto the disk surface, the disks were carefully transferred to an incubator maintained at 5% CO_2_ and 34°C for 2 h. Subsequently, additional growth medium was added to fully immerse the disks, which were then returned to the incubator and cultured for 3 days, with a media change at 48 h. Cell viability was assessed using the alamar-Blue assay (Invitrogen, Carlsbad, CA) according to the manufacturer's instructions and measured with a fluorescence microplate reader (FLx800, BioTek, Winooski, VT). All data are reported as percentage viability normalized to the FAp control. To confirm initial bacterial adhesion to the apatite surfaces, we performed additional adhesion studies after a 2-h incubation period. The disks were washed in PBS, then subjected to standing cell count analysis.

Following the alamarBlue assay, cells were fixed with 10% formalin (Epredia, Kalamazoo, MI), permeabilized with 0.3% Triton-X 100, blocked using a universal blocking solution (B10710, Thermo Fisher Scientific, Waltham, MA), and incubated overnight in primary antibodies for antiosteopontin (OPN; 1:100, ab8448; Abcam, Cambridge, MA) and antiosteocalcin (OCN; 10 μg/mL, MAB1419, R&D Systems, Minneapolis, MN). After washing away excess primary antibodies with PBS, the samples were incubated with fluorescently labeled secondary antibodies Alexa Fluor 647 Donkey anti-Rabbit at 10 μg/mL (A31573, Thermo Fisher Scientific, Waltham, MA) and Alexa Fluor 488 Goat anti-Mouse at 10 μg/mL (A11001, Thermo Fisher Scientific, Waltham, MA) for 1 h and protected from light. The samples were subsequently washed with PBS, stained with 4′,6-diamidino-2-phenylindole (DAPI; Thermo Fisher Scientific, Waltham, MA), cover-slipped with Fluoroshield (Abcam, Cambridge, UK), and imaged using a confocal microscope (Zeiss 700; Oberkochen, Germany).

### 2.5 | In Vivo Pilot Study: Tibial Defect Infection and Repair Model

Three male Lewis rats (6–9 weeks old; 386 ± 82 g) were used in accordance with an approved Institutional Animal Care and Use Committee (IACUC; University of Utah #2247) protocol. Animals were anesthetized and maintained using 3%–5% isoflurane in oxygen during the procedure. The right hind limb was shaved and prepared for sterile surgery using alternating scrubs of povidone-iodine and ethanol, followed by the IACUC-approved analgesic regimen. A 5 mm incision was made below the knee joint to access the medial tibial metaphysis. A cylindrical bone defect (3 mm diameter × 4 mm depth) was created using a sterile standard high-speed air-turbine dental handpiece and #8 carbide round bur (SS White Dental, Brussels, Belgium). A collagen foam (Foundation, J. Morita, Osaka, Japan) loaded with *S. aureus* (10^5^ CFU in PBS) was inserted into the defect and sealed with bone wax (Medline, Northfield, IL) to localize the bacterial inoculum within the bone defect. The incision was closed with absorbable 5–0 coated Vicryl sutures (Ethicon, Raritan, NJ). The rats were recovered and allowed unrestricted ambulation for 3 weeks to permit infection development. At 3 weeks postinoculation, a second surgery was performed under the identical anesthesia, analgesia, and aseptic conditions. The defect site was re-exposed, and incision line, soft tissue, and defect cavity swabs (Puritan, Guilford, ME) were collected to confirm infection. The defect was debrided and irrigated thoroughly with sterile PBS. The resulting enlarged defect was repaired using ~0.1 g of sintered 0.50 mol% CuFAp (*n* = 1) or FAp (*n* = 1) particles (250–425 μm, sintered at 1150°C). Again, the graft material was secured with bone wax (Medline, Northfield, IL). The incision was closed with 5–0 coated Vicryl sutures (Ethicon, Raritan, NJ), and animals were returned to unrestricted cage activity and monitored for an additional 12-week period.

At the study endpoint, the animals were euthanized. Tibiae were harvested, fixed in 10% Neutral Buffered Formalin (Epredia, Kalamazoo, MI) for 78 h. After tissue fixation, the harvested specimens underwent imaging with micro-computed tomography (μCT; Quantum GX2, PerkinElmer Health Sciences, Waltham, MA, USA) at scanning parameters 80 kV and 100 μA with an isotropic voxel size of 2.86 μm. Three-dimensional re-constructions were subsequently generated and analyzed to quantify new bone formation with Materialize Mimics software (Version 20.0, Leuven, Belgium).

After μ-CT imaging, specimens were processed for embedding in polymethyl methacrylate (PMMA). Sections were cut, ground, and polished, and then stained using Sanderson's Rapid Bone Stain (DHM, Loxley, AL). Images were acquired using a brightfield microscope (VHX-6000, Keyence, Osaka, Japan) and prepared for publication quality in Adobe Photoshop (Adobe Inc., San Jose, CA).

### 2.6 | Statistical Analysis

All data reported as mean±standard deviation (SD). For the bacterial adhesion comparison, CFU counts of bacteria adhered to the analyzed surfaces were compared across material groups (FAp and CuFAps) and bacterial strains (*S. aureus* and *P. aeruginosa)* using a two-way analysis of variance (ANOVA), followed by Tukey's post hoc test. For cytocompatibility data, the normalized cell viability percentages were compared between material groups (FAp and CuFAps) using one-way ANOVA, with Tukey's post hoc test for pairwise comparisons. All statistical analyses were performed using Stata Statistical software (Release 18, StataCorp LLC, College Station, TX). A *p* < 0.05 was considered the threshold for statistical significance.

## 3 | Results

Although characterization was not the primary focus of this study, it was essential to confirm the composition of the in-house synthesized materials. Thus, the as-synthesized powders were characterized to confirm their structure and purity.

**FIGURE 1 |:**
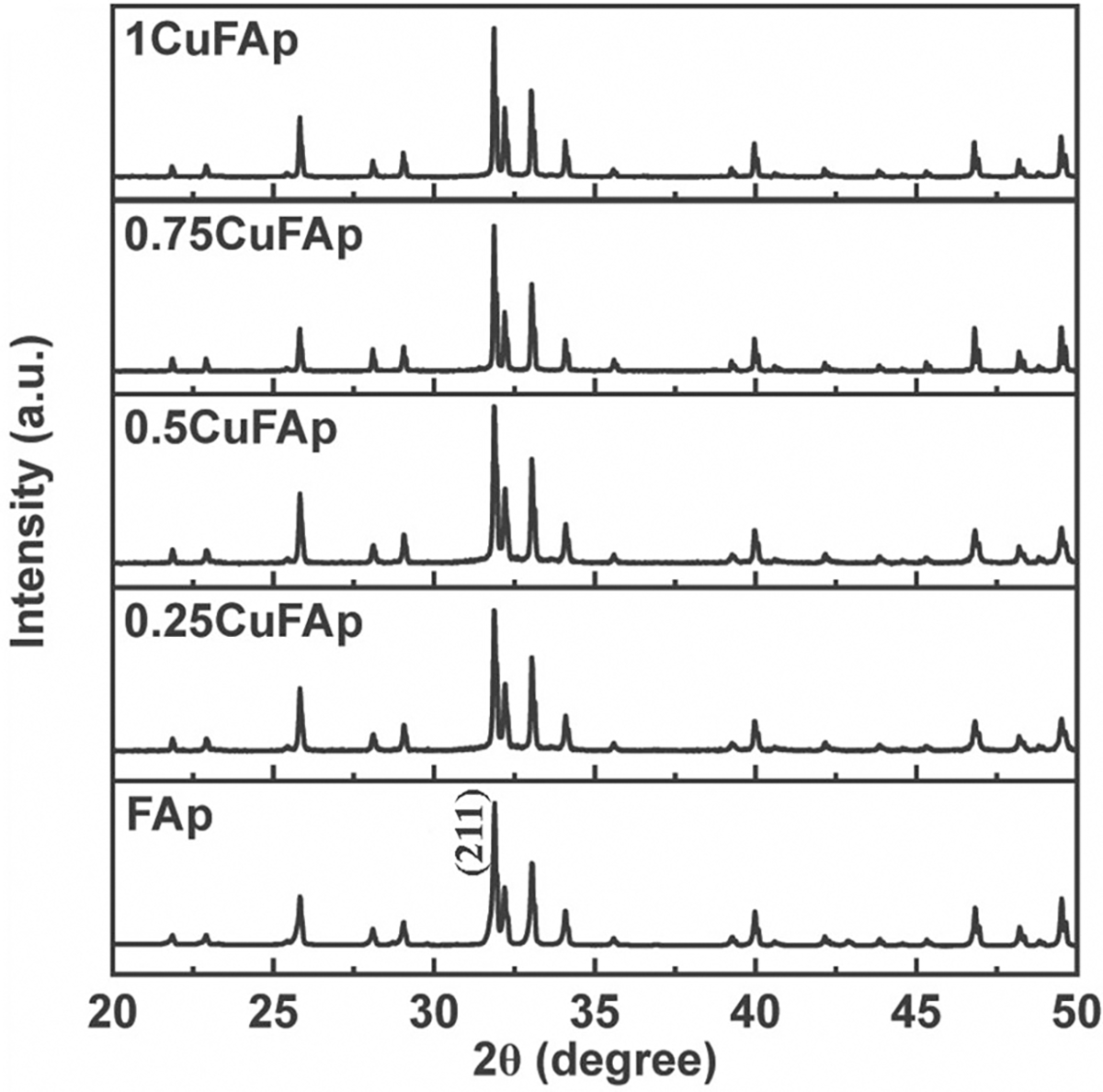
Representative X-ray diffraction (XRD) patterns of the in-house synthesized undoped and low-percentage Cu-doped FAp samples, highlighting the major diffraction peaks within the 2*θ* range of 20°–50°.

These diffractograms were initially used to identify the phases and crystal structures of FAp and CuFAp samples. Figure 1 presents the diffractograms (20°–50°) of FAp and 0.25–1.0 CuFAps. All peaks aligned with the reference FAp patterns, where the Weighted-Profile R-factor (Rwp) for the FAp reference and synthesized powder was less than 10 for all datasets. Characteristic peaks for FAp observed at 2*θ* values of 25.84°, 31.92°, and 32.29° correspond to the (002) basal plane, (211) and (112) diagnostic, and (300) characteristic of hexagonal phase reflection planes, which were consistent with the P6_3_/m symmetry of FAp. CuFAps, compared to undoped FAp, exhibited similar peak positions, confirming the preservation of the hexagonal structure across all doping levels. To further investigate the efficacy of the Cu dopant on the FAp structure, the lattice parameters, unit cell volume, and crystallite size were determined and are given in Table 1.

The crystallite sizes (*D*_c_s) of FAp and CuFAp samples (Table 1) were determined using the Debye–Scherrer equation, based on the full width half maximum (FWHM) of the (211) diffraction peak. The data indicated that crystal size increased progressively with increasing Cu doping, rising from 71.21 nm for undoped FAp to 78.18 nm in 1 CuFAp. A slight sharpening of the peak and a reduction in FWHM were observed at a higher doping concentration of 1 mol%.

**FIGURE 2 |:**
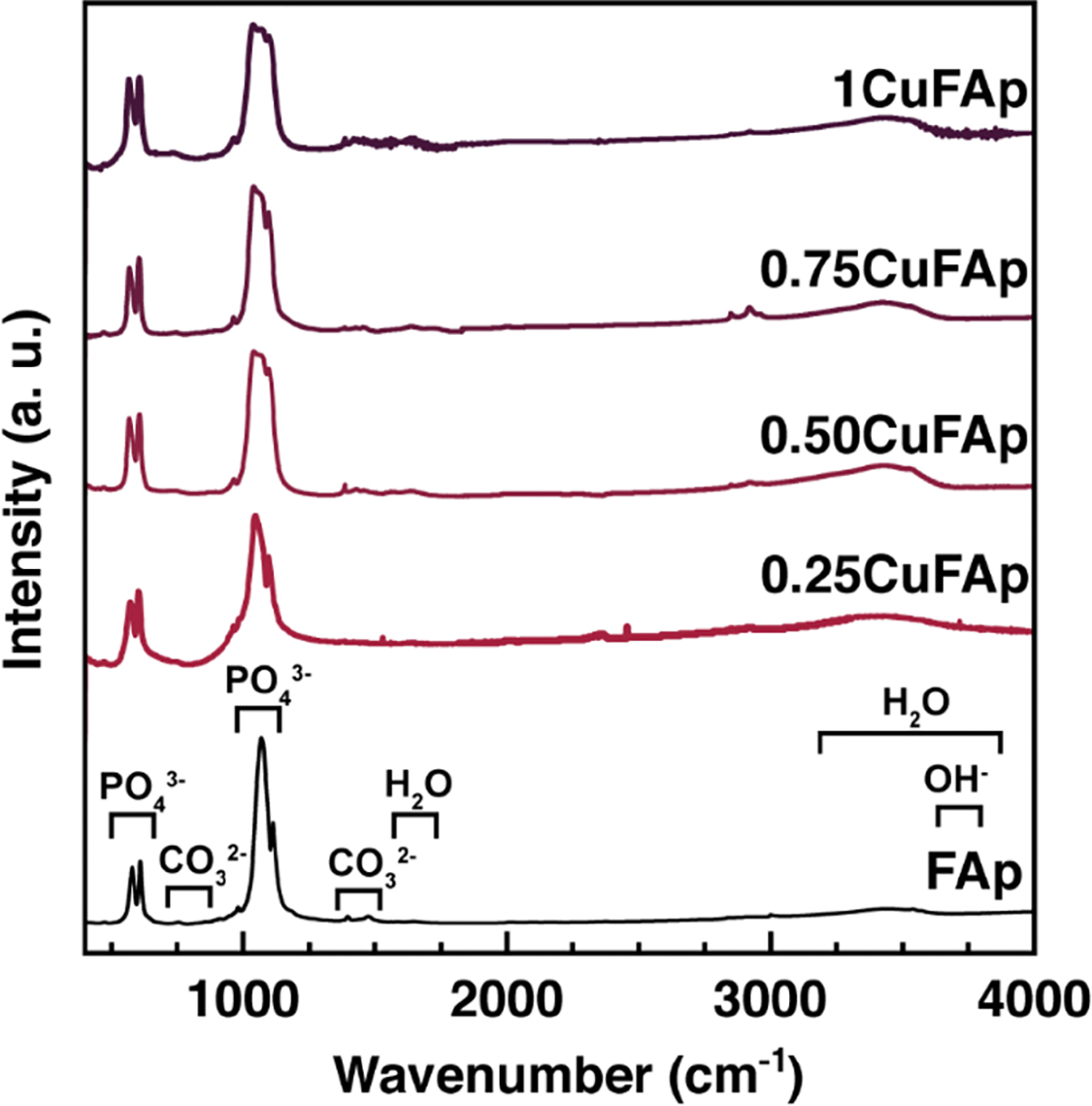
A representative set of FTIR spectra shows characteristic phosphate peaks at 560–565, 600–610, 960–962, and 1040–1090 cm^−1^.

The FTIR spectra are given in Figure 2, confirming the characteristic phosphate (PO_4_^3−^) peaks in the regions of ~1090, ~1050, ~570–600, ~478, and ~960 cm^−1^. Notably, a shift of phosphate peaks to higher wavenumbers, accompanied by peak broadening, was observed with the increasing Cu concentration. In CuFAp samples, low intensity secondary peaks, such as those of carbonates (CO_3_)^2−^ (~1420–1500 cm^−1^) were observed. Although no sharp OH^−^ stretching band was detected at ~3570 cm^−1^ in CuFAps, a broad, low-intensity absorption band at 3200–3700 cm^−1^, representing the adsorbed water molecules, was observed in all CuFAp samples.

ICP–OES analysis was conducted to quantify the elemental composition. The results are shown in Table 2 present the molar concentrations of ions relative to their theoretical values. In FAp, the Ca/P molar ratio was 1.68, aligning well with the theoretical value of 1.67. In CuFAp, Cu was confirmed, with measured concentrations matching the target doping levels. The (Ca + Cu)/P molar ratio remained between 1.66 and 1.70, indicating the probable incorporation of Cu into the FAp structure throughout the doping range studied.

**FIGURE 3 |:**
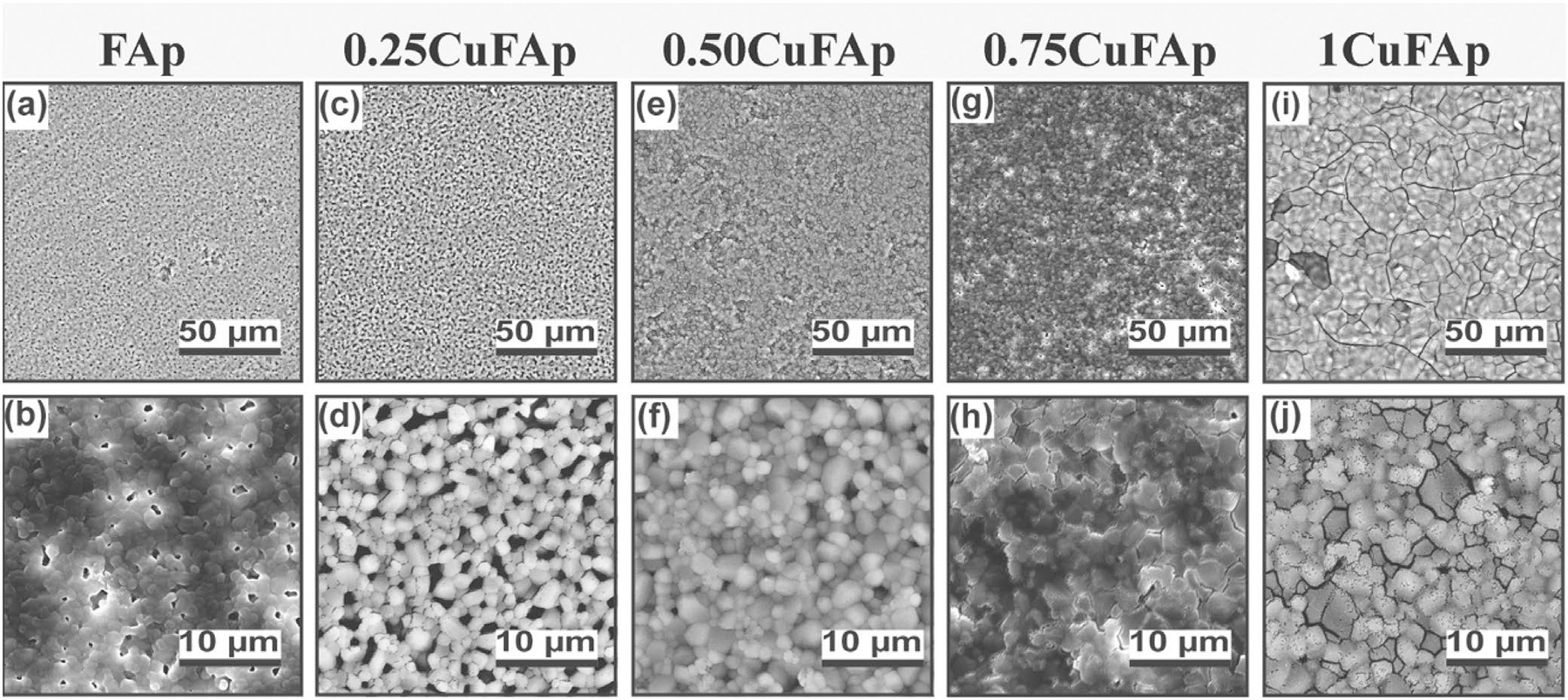
A set of micrographs showing the topography and morphology of sintered FAp (a and b), 0.25 CuFAp (c and d), 0.50 CuFAp (e and f), 0.75 CuFAp (g and h), and 1 CuFAp (i and j). Scale bars: Top row, 50 μm. Bottom row, 10 μm.

SEM was used to examine the surface morphology and microstructure of FAp and CuFAp samples sintered at 1150°C (Figure 3). Grain size measurements obtained from the SEM images are summarized in Table 3. The FAp sample exhibited a relatively smooth surface with uniform grain size and porosity distributions. In contrast, the CuFAp samples exhibited greater grain-size variability, with the highest Cu doping level (1 CuFAp) showing the greatest variation and layer separation.

**FIGURE 4 |:**
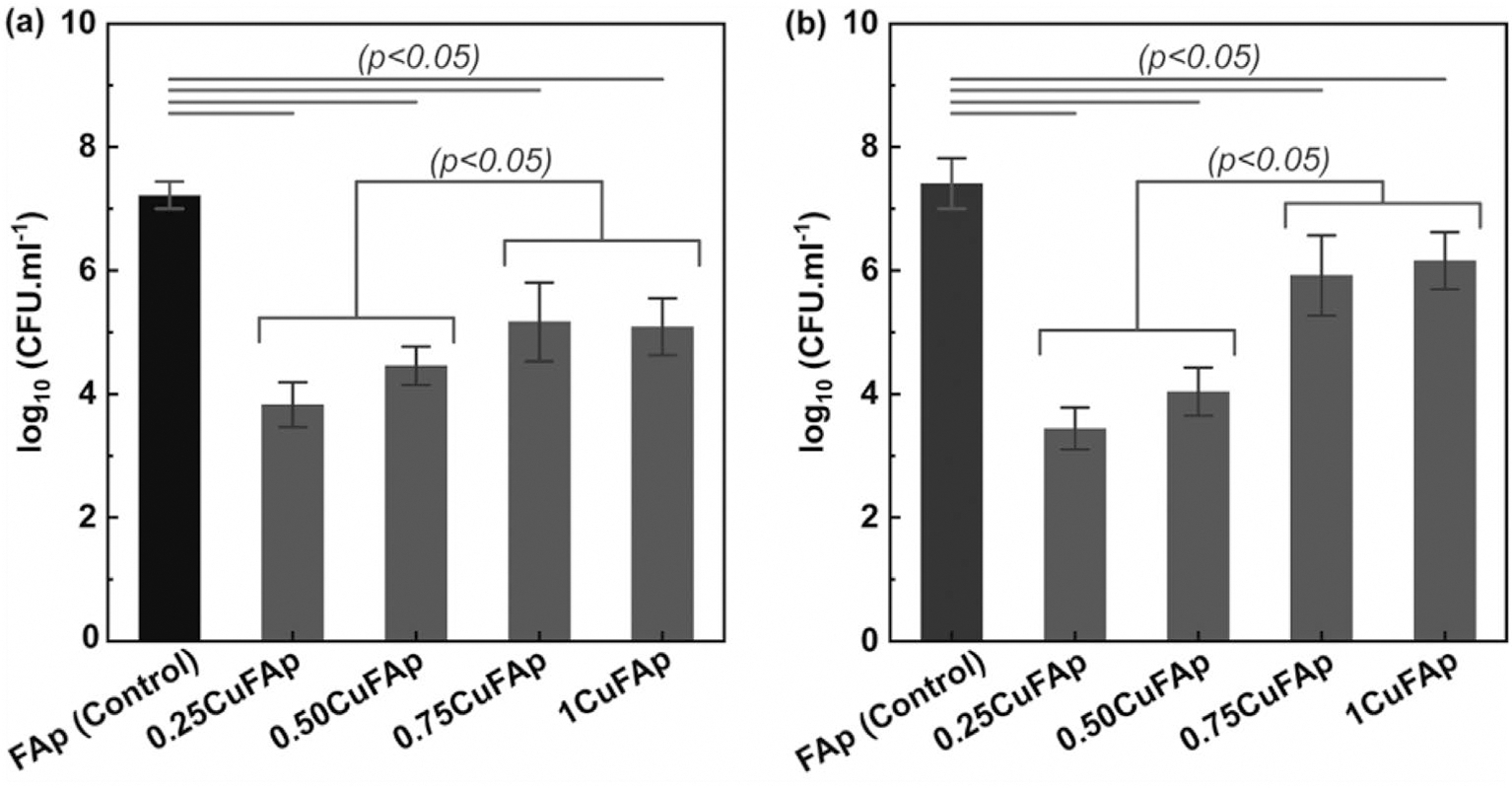
A set of bar charts showing the number of adhered (a) *S. aureus* and (b) *P. aeruginosa* on FAp and CuFAp surfaces after 2 h of static incubation with an initial inoculum of 10^8^ CFU/cm^2^. Error bar = standard deviation. The *Y*-axis showed log-fold reduction.

As described in the Methods section, differences in bacterial adhesion on these apatite surfaces were analyzed. Figure 4 illustrates the reduced *S. aureus* and *P. aeruginosa* bacterial adhesion properties of CuFAp surfaces compared to the undoped control, FAp, surface. Cu doping reduced bacterial adhesion across all tested concentrations (*p* < 0.05). The 0.25 CuFAp and 0.50 CuFAp surfaces demonstrated the most pronounced effects, with approximately a 3-log reduction in bacterial adhesion compared to FAp. In contrast, the 0.75 CuFAp and 1 CuFAp surfaces showed a moderate yet statistically significant 2 log-fold reduction (*p* < 0.05). Additionally, two-way ANOVA indicated that bacterial species did not significantly affect adhesion outcomes (*p* > 0.05).

**FIGURE 5 |:**
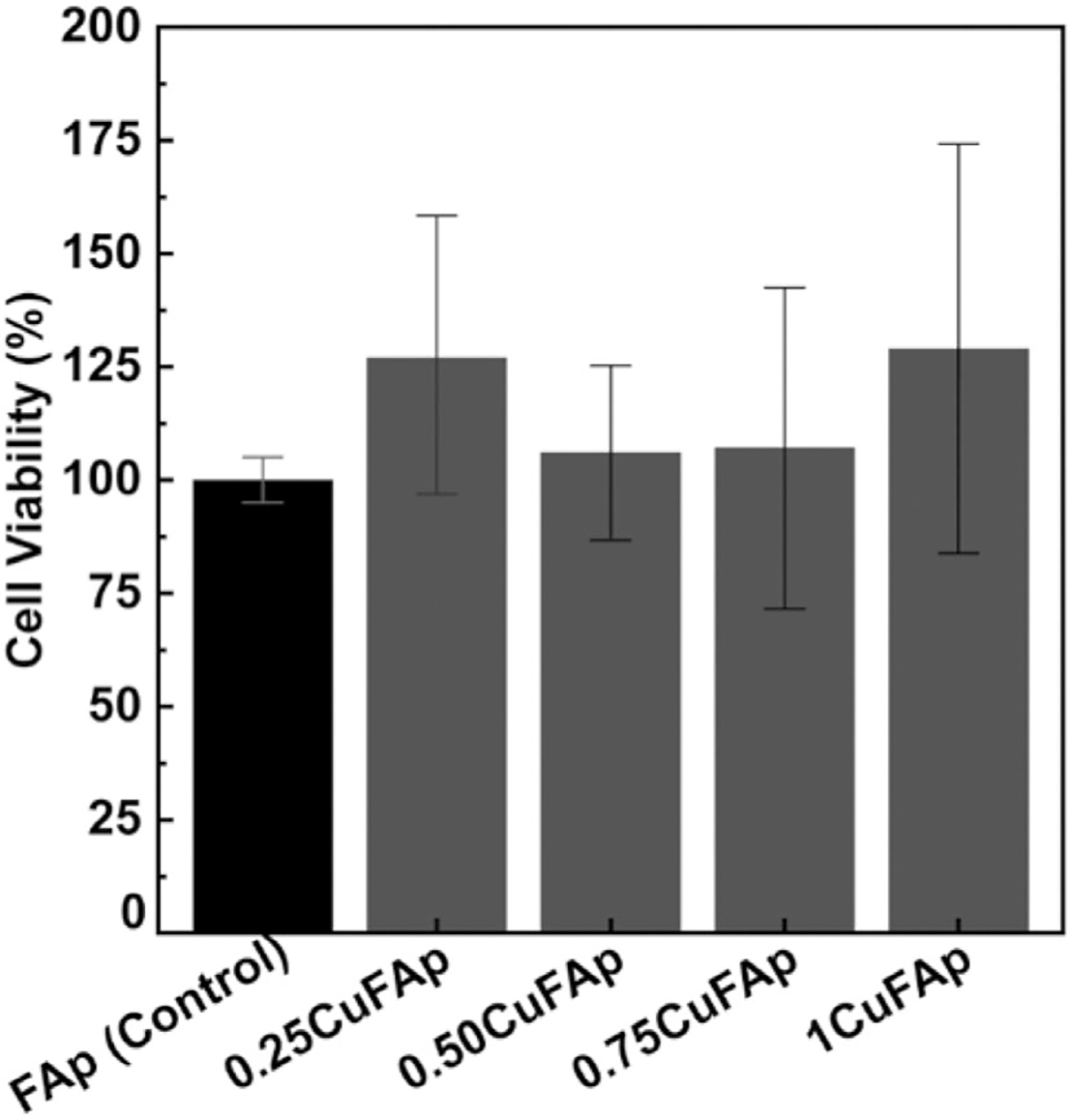
A bar chart showing comparative cell viabilities assessed using alamarBlue at the 3-day time point, normalized to the FAp (control) group, with no statistical significance among all groups. Error bars = standard deviation.

**FIGURE 6 |:**
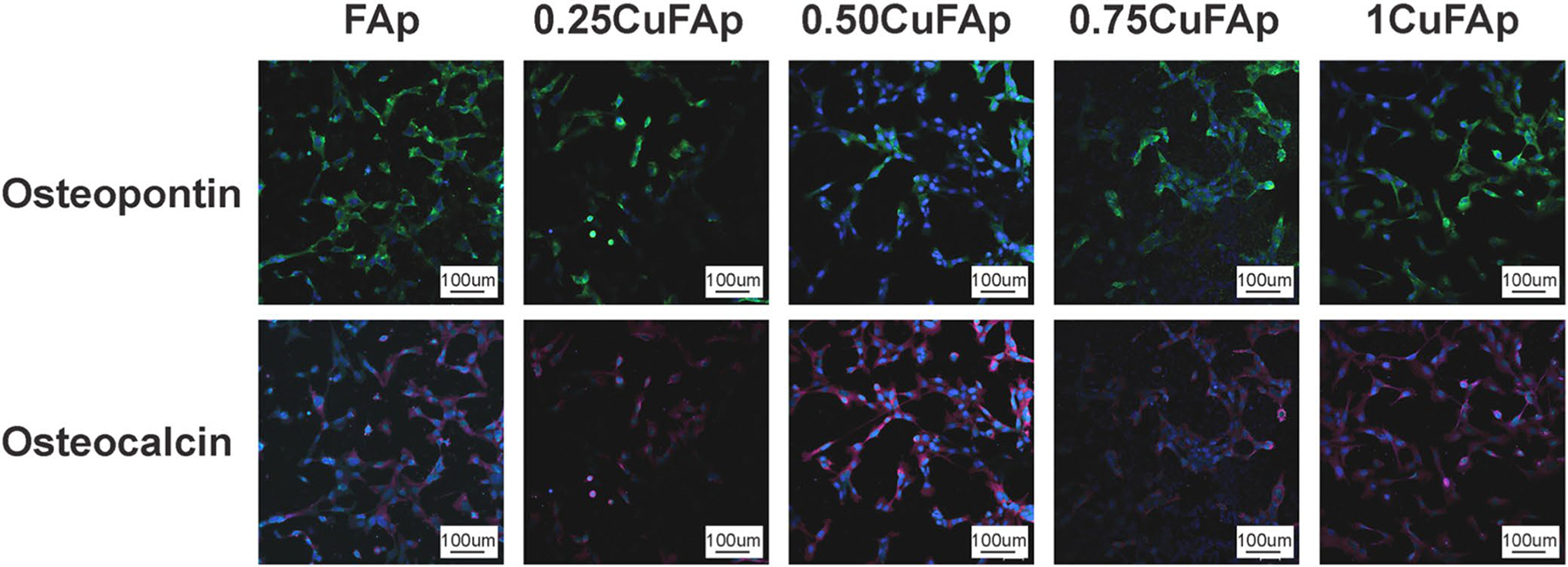
A set of confocal images showing immunofluorescence markers in osteoblasts cultured on FAp and CuFAp surfaces after a 3-day period. Cell nuclei are stained blue with DAPI; osteopontin in green (top), and osteocalcin also in red (bottom). Scale bar is 100 μm in all images.

The effects of the surfaces on osteoblast adhesion and differentiation were assessed 3 days postseeding (Figure 5). There were no significant differences found in cell viability among the different surfaces (Figure 5; *p* > 0.05). Additionally, it is worth mentioning that at 2 h, ~80% of the inoculated osteoblasts had adhered to the surface. Confocal microscopy images (Figure 6) showed comparable cell densities across all surfaces. The cells exhibit elongated morphologies typical of healthy, viable cells. There were no significant differences in the expression levels of osteopontin or osteocalcin; all surfaces showed positive expression of these bone-forming markers.

All rats in the pilot animal study showed clinical signs of local inflammation at the time of the second surgery. The presence of purulence did not appear to affect mobility, and was present in all animals. Swabs and tissue samples collected during the second surgical procedure confirmed bacterial cultures (semiquantitative data; four-quadrant streaking methods produced confluent growth (4+ growth)), confirming infection. Twelve weeks after debridement and repair with apatite granules, there were no visible signs of infection at the defect sites. Tissues collected at necropsy were free of culturable bacteria (only 10–20 CFU growth).

**FIGURE 7 |:**
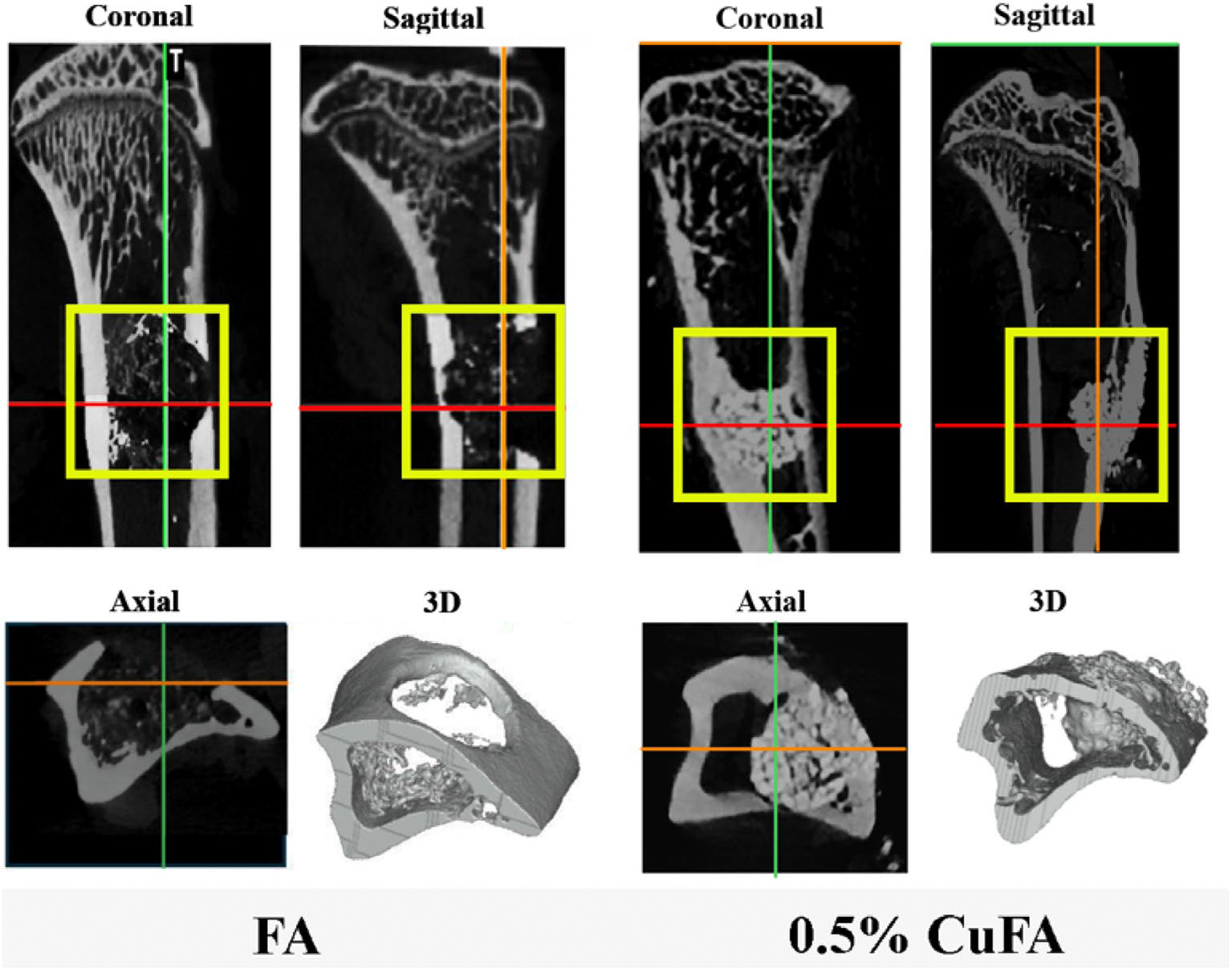
Top row: A representative set of μ-CT images of rat tibial defects following infection induction, subsequent surgical debridement, and repair surgeries using apatites as scaffolds. The defects were filled with either FAp (Right) or 0.50 CuFAp (left) granules and evaluated at 12 weeks postimplantation. Bottom row: 3D reconstructed images of the defect sites using μ-CT data. The images show new bone formation within the surgically created defect sites (gray).

**FIGURE 8 |:**
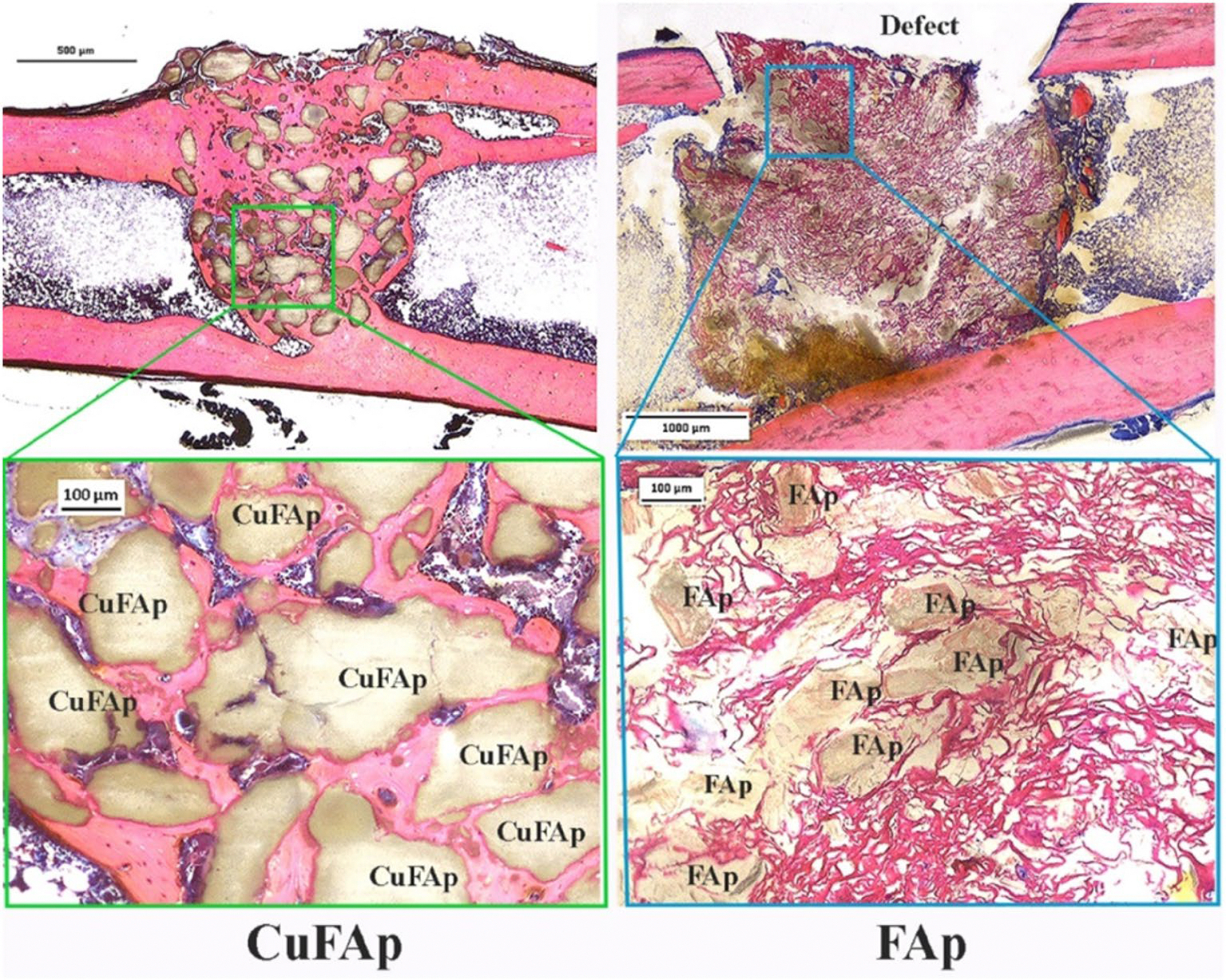
Representative sagittal rat tibial sections at 12 weeks postrepair with apatite granules, stained with Sanderson's Rapid Bone Stain, showing the repaired surgical defect sites. Left: defect repaired with 0.50 CuFAp; Right: defect repaired with FAp. Pink indicates bone. Off-white indicates apatite granules. Bridges consist of apatite granules and new bone; blue represents nuclei or soft tissues.

Figure 7 presents representative μ-CT images of repaired tibial defects following infection induced with 10^5^ CFU of *S. aureus* 4 weeks prior, and subsequent treatment with either FAp or 0.5 CuFAp granules, then the animals were observed for 12 weeks. These images reveal clear differences in bone regeneration between the two treatment groups. In the FAp-treated group, the defect margins remain distinct, with incomplete bone formation and an absence of cortical bridging across the defect site, indicating limited regenerative capacity under infected conditions. In contrast, the 0.50 CuFAp-treated group showed substantial new bone formation, with evident bridging of the defect and restoration of cortical continuity. This observation was validated by the histology images given in Figure 8.

Figure 8 presents representative histological sections of tibial defects treated with CuFAp and FAp. In the 0.50 CuFAp group, abundant new bone formation was observed surrounding the granules, with direct bone apposition and intimate integration between the material and host tissue. No histological signs of acute or chronic inflammation or fibrous encapsulation were evident. The newly formed bone displayed a mature lamellar structure and successfully bridged the infected cortical defect. In contrast, the FAp-treated group shows limited bone regeneration, both along the cortical wall and within the medullary canal. The cortical defect remained unbridged, and the bone formed around the FAp granules appeared sparse and disorganized, woven, lacking the mature lamellar architecture seen in the CuFAp group.

## 4 | Discussion

This study successfully synthesized and characterized a series of CuFAps and evaluated their antibacterial adhesion properties and cytocompatibility. The findings support the hypothesis that CuFAp surfaces doped with 0.25%–1% Cu significantly reduced the adhesion of *S. aureus* and *P. aeruginosa* (*p* < 0.05) on their surfaces, while maintaining osteoblast viability (*p* > 0.05) and preserving bone regenerative potential, as demonstrated in a follow-up pilot study involving an infected defect site in rats. In summary, the data indicated that Cu-doped surfaces containing less than 0.50 mol% Cu exhibit effective bacteriostatic properties without compromising bone regenerative capacities.

Although the primary objective of this study was to evaluate the bacteriostatic properties and biocompatibility of CuFAp, material characterization was essential to confirm phase purity and verify successful Cu incorporation at the intended doping levels. Therefore, FTIR and XRD analyses were conducted to verify the purity of the apatite phase. The results confirmed the successful synthesis of the intended apatites (Figures 1–3). Specifically, FTIR data verified that the characteristic phosphates (PO_4_^3−^) peaks of apatite appear at ~1090 and ~1050 cm^−1^ (P–O asymmetric stretching) and bending doublets at ~560–600, 420–478, and ~960 cm^−1^ (P–O symmetric stretching). The absence of a sharp OH^−^ peak at ~3570 cm^−1^ (OH^−^ stretching) in FAp confirmed the complete substitution of both hydroxyl groups. However, in this region (3200–3700 cm^−1^), a broad peak was apparent in CuFAp samples. The intensity of this band increased with increasing Cu dopant content. It is likely that the presence of additional Cu—either incorporated within the apatite lattice or present in trace copper oxide—enhanced the hygroscopicity of the CuFAp samples, resulting in the observed broad band indicative of adsorbed water molecules, which has been reported in Cu-doped FAp and also HAp materials [40], but the intensity of the band was weak. Additionally, such peaks were commonly reported in FTIR spectra of FAp synthesized via aqueous precipitation methods [40]. Notably, a previously published study on the hydrothermal processing of CuO with FAp did not report a similar broad band, suggesting a potential absence of CuO in our in-house–synthesized CuFAp [39]. This interpretation is further supported by the absence of a Cu–O vibrational band in the 510–520 cm^−1^ region [72].

The XRD analysis revealed a highly crystalline material with over 85% crystallinity and no detectable secondary calcium phosphate phases, such as β-TCP, a common impurity in apatite synthesis processes [73]. This result is consistent with the previous literature, which indicated the absence of β-TCP byproduct with lower Cu doping [40, 57]. Additionally, Rietveld refinement—an iterative process by starting from the initial structures parameters and then adjusting scale factors, zero shift, lattice parameters, peak profile parameters, and atomic positions to optimize the fit—of the XRD data (Table 1) indicated shifts in lattice parameters with increasing Cu substitutions, likely due to the substitution of Ca^2+^ ions with Cu^2+^ ions, which have a smaller ionic radius (0.73 Å) than Ca^2+^ (1.00 Å) [74]. These observations are also supported by prior studies on other Cu-doped apatites [40, 50, 51, 75]. Additionally, the observed increase in crystallite size and peak sharpening with increasing Cu doping may be attributed to the role of Cu^2+^ ions in enhancing crystallization behavior, as previously reported for HAp [76]. It is believed that Cu incorporation could affect nucleation and subsequent crystal growth by promoting a more stable and ordered lattice arrangement. While similar lattice parameters have been reported for Cu-doped FAp at low doping levels [40], existing literature on Cu-doped HAp indicates that higher Cu doping levels decrease crystallite size [41, 77–79], which contrasts with the findings of the present study and requires further investigation.

The ICP-OES data were used to quantify the Cu content and verify that the (Ca + Cu)/P molar ratio remained close to 1.67 (ranging from 1.65 to 1.68), which is characteristic of stoichiometric apatite. This value falls within the acceptable range of 1.5 < *x* < 1.9 for nonstoichiometric FAp [80, 81], indicating that Cu^2+^ incorporation within the studied range did not significantly disrupt the overall cation-to-phosphate ratio.

In our study, the microbiological evaluation of lower (0.25–1 mol%) concentrations of CuFAps demonstrated reduced bacterial adhesion while supporting the attachment and proliferation of osteoblasts (Figure 4), supporting previous studies [40, 65]. These findings also align with previous studies, which report that Cu-doped HAp exhibits enhanced bacteriostatic activity against a range of pathogens, including *S. aureus*, *S. epidermidis*, *P. aeruginosa*, and *E. coli*, when compared to an undoped control [40, 51, 82, 83]. To date, most existing research has focused on the bacteriostatic efficacy of Cu-doped HAp [50, 84–88], with comparatively limited attention given to FAp [40]. Above published CuFAp data exhibited greater efficacy in reducing both Gram-positive *S. aureus* and Gram-negative *Escherichia coli* (*E. coli*) colony formation, at doping levels ranging from 0.5 to 20 mol, and the direct comparison of CuFAp to CuHAp showed greater antimicrobial activity in CuFAp, with CuHAp showing only limited inhibition of *E. coli* [40, 89, 90]. Specifically, 0.25 CuFAp and 0.50 CuFAp significantly inhibited, by approximately 2-log fold reduction, the initial adhesion of *S. aureus* and *P. aeruginosa*, major pathogens implicated in orthopedic bone infections [91–93]. While most prior work has examined Cu-doped apatites in their as-synthesized powder form [40, 50, 85], or as a coating on metal surfaces [51, 87], we evaluated sintered CuFAp bulk bodies engineered for bone scaffold applications. Despite differences in material preparation and structure, our sintered CuFAp surfaces limited bacterial adhesion to levels comparable to or better than those observed in other Cu-doped apatite studies [40, 41, 76, 94, 95]. However, the threshold concentration of Cu required to achieve a bactericidal effect remains ambiguous in the literature. While some studies suggest that Cu concentrations exceeding 5 mol% are necessary to significantly reduce bacterial viability in HAp systems [76], others have reported that even lower concentrations (~1–2 mol%) can effectively prevent biofilm formation through surface-mediated interactions rather than direct bacterial killing [86]. Our data further lowers these effective concentrations to 0.25–0.50 mol% Cu doping.

As reported above, although the antimicrobial properties of CuFAp have been documented, the mechanism underlying this effect remains less well understood. The observed antibacterial and surface antiadhesion properties could be attributed to both chemical and physical factors, which likely act synergistically to enhance the antiadhesion and bacteriostatic performance of CuFAp. It is known that surface microtopographic patterns can either promote or inhibit bacterial adhesion, depending on how closely the surface features match the bacteria's size and shape [96–99]. Likewise, within the apatite literature, studies have shown that grain size can influence both osteoblast behavior and bacterial adhesion [100–102]. For example, our results show that increasing Cu content in FAp was associated with increased average grain size and greater grain-size variability (Table 3). Although the bacterial attachment data showed reduced adhesion on 0.25 and 0.5 CuFAp surfaces with smaller grain sizes (1.15–1.24 μm), greater bacterial adhesion was observed on FAp surfaces with an even smaller grain size (0.75 μm). This observation suggests that grain size alone is unlikely to be the determining factor. Another possible mechanism is Cu ion release, which would have been a plausible mechanism given the well-established antibacterial properties of Cu ion. At the doping levels used in this study, it is unlikely to be a primary contributor, as discussed in our previous publication [65]. Thus, other surface characteristics altered by Cu ion incorporation, such as surface roughness, zeta potential, and surface energy, are more likely to contribute, in part, to the observed effects. Future work should systematically characterize these surface properties to better understand the mechanisms by which Cu doping in FAp influences bacterial and cell adhesion.

While bone graft substitutes endowed with bacteriostatic properties would provide an advantage in infection-prone environments, these materials must also retain cytocompatibility and osteoconductivity. If excessive concentrations of Cu ions were released in vivo, they could induce oxidative stress and impair cellular function, ultimately leading to the apoptosis of host cells [49]. Thus, evaluating both cytocompatibility and osteoconductivity of these materials became essential to this study. Cell viabilities were assessed in vitro using the alamarBlue assay, while osteoconductivity was evaluated using osteogenic markers, including OPN and OCN. The results (Figures 5 and 6) indicated that low percent Cu incorporation did not adversely affect osteoblast viability at these doping levels. There were no statistically significant differences observed between Cu-doped groups and the undoped FAp control, suggesting that Cu levels up to 1 mol% did not appear to induce cytotoxicity to osteoblasts. These findings are consistent with previous reports on Cu-doped HAp systems [103]. For example, Noori et al. [76] demonstrated that, at 2 mol%, Cu-doped HAp did not impair osteogenic potential or endothelial cell migration; however, their study did not investigate concentrations below 1 mol%. In another study, 0.5% Cu-doped HAp coatings promoted osteogenic differentiation, but at higher levels, cellular metabolic activity was limited [49, 85, 104]. Taken together, these findings supported the notion that excessive Cu can trigger cytotoxic effects through mechanisms such as oxidative stress and mitochondrial dysfunction [105]. In contrast, doping levels below 1 mol%, as presented in this study, appeared to maintain cytocompatibility and promote osteogenic activity.

The pilot animal data further confirmed the 0.50 CuFAp's ability to repair bone defects within infected, critical-size defects, but this data needs to be confirmed in a statistically powered infected animal cohort. Although no quantitative histomorphometric measurements were performed due to the pilot nature of this study, the qualitative findings support the osteoconductive and nonimmunogenic properties of the 0.50 CuFAp bone substitute. The fact that bone tissue formed in apposition to granules demonstrated seamless integration of bone with the CuFAp, without fibrous encapsulation. These findings suggest that CuFAp supports bone regeneration within the bacterially contaminated sites.

This study has several limitations that warrant further investigation to fully assess the long-term effectiveness and clinical applicability of less than 1% doped CuFAp materials. Our results demonstrate promising initial antibacterial adhesion while maintaining biocompatibility; however, the durability of these effects over extended time remains unknown. It is essential to evaluate whether Cu ion release remains within biologically safe limits during prolonged time in situ. Moreover, given the broad range of pathogens implicated in orthopedic and dental infections, future work should assess the bacteriostatic efficacy of CuFAp against a broader spectrum of clinically relevant microorganisms, including fungi. Additionally, it is acknowledged that the observed decrease in microbial adhesion to CuFAp may be influenced by its grain size [100, 106]. Thus, future studies should need to control for this confounding variable. Incorporating biofilm reactor-based studies that simulate dynamic physiological conditions would provide more realistic insights into the performance of these materials. Quantitative analysis of surface properties, such as wettability and surface energy, is also necessary, as these factors play a critical role in modulating cell adhesion, proliferation, and overall bioactivity. As previously noted, the precise mechanisms underlying the antibacterial behavior of CuFAp—whether related to ion release, surface chemistry, or microstructure—remain unclear and merit focused studies. Additionally, the influence of sintering temperatures on microstructure, ion release, and antibacterial performance was not explored in this study and should be systematically investigated. Ultimately, comprehensively powered in vivo studies will be essential to validate the biocompatibility, bacteriostatic efficacy, resorption properties, and long-term safety of CuFAp in physiological environments and to determine its true potential for clinical translation in bone replacement and infection-prone orthopedic applications. It is also important to note that the small size of rat bone limits the defect size that can be safely created without compromising structural integrity. Based on our small animal data presented here, further investigation of CuFAps should be conducted in larger, clinically relevant defect models [107–109] to better assess their therapeutic potential, perhaps even using the biofilm as inoculum [110].

In summary, this study demonstrated that low-level (0.25–0.5 mol%) Cu-substituted FAp, synthesized via wet chemical precipitation, effectively reduced bacterial adhesion while supporting osteoblast adhesion, differentiation, and bone deposition. Structural analyses confirmed successful Cu incorporation without the formation of secondary phases, and microstructural observations suggest that finer grain sizes may enhance bacteriostatic performance. These findings highlight CuFAp as a promising biomaterial for bone replacement and regeneration, particularly in contaminated sites, thus warranting further in vivo validation.

## 5 | Conclusion

These data support the potential of 0.25–0.50 mol% Cu-doped FAps as multifunctional biomaterials for dental and orthopedic applications. Because Cu is incorporated into the lattice structure, the antibacterial adhesion properties of these materials are expected to persist throughout the lifetime of the scaffold, rather than exhibiting the short-lived burst effect typically observed when antibiotics are blended with grafts. Moving forward, these materials can be effectively utilized in various forms—including pastes, scaffolds, and granules—for bone regeneration applications such as periodontal defect repair, bone augmentation, and implant site preservation.

## Figures and Tables

**TABLE 1 |:** Calculated lattice parameters and related properties of FAp and CuFAps from the diffractograms.

Sample ID	*a* (Å)	*c* (Å)	*V* (Å^3^)	*D*_c_ (nm)

FAp	9.36	6.88	524.20	71.21
0.25 CuFAp	9.36	6.88	524.20	73.32
0.50 CuFAp	9.35	6.88	522.19	74.24
0.75 CuFAp	9.35	6.88	522.12	73.67
1 CuFAp	9.34	6.88	521.59	78.18

*Note:* Lattice parameters *a*, *c*, unit cell volume (*V*), and crystallite size (*D*_c_).

**TABLE 2 |:** Elemental compositions of the studied apatites as determined by inductively coupled plasma optical emission spectrometry (ICP–OES).

	(Ca + Cu):P		Cu molar%	
		
Sample ID	Measured	Theoretical	Measured	Theoretical

FAp	1.68	1.67	0	0
0.25 CuFAp	1.69	1.67	0.20	0.25
0.50 CuFAp	1.66	1.67	0.60	0.50
0.75 CuFAp	1.68	1.67	0.70	0.75
1 CuFAp	1.70	1.67	1.10	1.00

**TABLE 3 |:** Grain size distribution. Average grain size ($\overset{-}{d}$), size range (±*σ*), and standard deviation (SD) of pure fluorapatite (FAp) and Cu-doped FAps (CuFAps).

Sample ID	$\overset{‾}{d}$ (μm)	±*σ* (μm)	SD (μm)

FAp	0.75	0.1	0.06
0.25 CuFAp	1.15	0.3	0.13
0.50 CuFAp	1.24	0.3	0.12
0.75 CuFAp	1.35	0.3	0.12
1 CuFAp	1.51	0.5	0.19

## Data Availability

The data that support the findings of this study are available on request from the corresponding author. The data are not publicly available due to privacy or ethical restrictions.

## References

[1] Trampuz A, Gilomen A, Fluckiger U, Frei R, Zimmerli W, and Widmer A, “141 Treatment Outcome of Infections Associated With Internal Fixation Devices: Results From a 5-Year Retrospective Study (1999–2003),” International Journal of Infectious Diseases 10 (2006): S79.

[2] Clauss M, Trampuz A, Borens O, Bohner M, and Ilchmann T, “Biofilm Formation on Bone Grafts and Bone Graft Substitutes: Comparison of Different Materials by a Standard In Vitro Test and Microcalorimetry,” Acta Biomaterialia 6, no. 9 (2010): 3791–3797. 20226886 10.1016/j.actbio.2010.03.011

[3] Jacobs A, Renaudin G, Forestier C, Nedelec J-M, and Descamps S, “Biological Properties of Copper-Doped Biomaterials for Orthopedic Applications: A Review of Antibacterial, Angiogenic and Osteogenic Aspects,” Acta Biomaterialia 117 (2020): 21–39. 33007487 10.1016/j.actbio.2020.09.044

[4] Fry DE and Barie PS, “The Changing Face of *Staphylococcus aureus*: A Continuing Surgical Challenge,” Surgical Infections 12, no. 3 (2011): 191–203. 21812657 10.1089/sur.2011.068

[5] Vogely HC, Oosterbos C, Puts E, , “Effects of Hydroxyapatite Coating on Ti-6Al-4V Implant-Site Infection in a Rabbit Tibial Model,” Journal of Orthopaedic Research 18, no. 3 (2000): 485–493. 10937638 10.1002/jor.1100180323

[6] Schmidt AH, “Autologous Bone Graft: Is It Still the Gold Standard?,” Injury 52 (2021): S18–S22. 10.1016/j.injury.2021.01.043 33563416

[7] Khan SN, Cammisa JFP, Sandhu HS, Diwan AD, Girardi FP, and Lane JM, “The Biology of Bone Grafting,” Journal of the American Academy of Orthopaedic Surgeons 13, no. 1 (2005): 77–86. 15712985

[8] Fillingham Y and Jacobs J, “Bone Grafts and Their Substitutes,” Bone & Joint Journal 98, no. 1_Suppl_A (2016): 6–9. 10.1302/0301-620X.98B.36350 26733632

[9] Baldwin P, Li DJ, Auston DA, Mir HS, Yoon RS, and Koval KJ, “Autograft, Allograft, and Bone Graft Substitutes: Clinical Evidence and Indications for Use in the Setting of Orthopaedic Trauma Surgery,” Journal of Orthopaedic Trauma 33, no. 4 (2019): 203–213. 30633080 10.1097/BOT.0000000000001420

[10] Betz RR, “Limitations of Autograft and Allograft: New Synthetic Solutions,” Orthopedics 25, no. 5 (2002): S561–S570. 12038843 10.3928/0147-7447-20020502-04

[11] Younger EM and Chapman MW, “Morbidity at Bone Graft Donor Sites,” Journal of Orthopaedic Trauma 3, no. 3 (1989): 192–195. 2809818 10.1097/00005131-198909000-00002

[12] Miron RJ, “Optimized Bone Grafting,” Periodontology 94, no. 1 (2024): 143–160. 10.1111/prd.12517 37610202

[13] Aijaz M, Ahmad M, Ansari MA, Ahmad S, and Kumar A, “Tools and Techniques Used for the Development of Scaffold for Bone Tissue Regeneration: A Detailed Review,” Biointerface Research in Applied Chemistry 14, no. 5 (2024): 123.

[14] Singh B, Dubey AK, Kumar S, Saha N, Basu B, and Gupta R, “In Vitro Biocompatibility and Antimicrobial Activity of Wet Chemically Prepared Ca_10-x_Ag_x_(PO_4_)_6_(OH)_2_(0.0≤x≤0.5) Hydroxyapatites,” Materials Science and Engineering: C 31, no. 7 (2011): 1320–1329.

[15] Bhadang K, Holding C, Thissen H, McLean K, Forsythe J, and Haynes DR, “Biological Responses of Human Osteoblasts and Osteoclasts to Flame-Sprayed Coatings of Hydroxyapatite and Fluorapatite Blends,” Acta Biomaterialia 6, no. 4 (2010): 1575–1583. 19857609 10.1016/j.actbio.2009.10.029

[16] Borkowski L, Jojczuk M, Belcarz A, , “Comparing the Healing Abilities of Fluorapatite and Hydroxyapatite Ceramics in Regenerating Bone Tissue: An In Vivo Study,” Materials 16, no. 17 (2023): 5992. 37687681 10.3390/ma16175992 PMC10488477

[17] Chen W, Wang Q, Meng S, , “Temperature-Related Changes of ca and P Release in Synthesized Hydroxylapatite, Geological Fluorapatite, and Bone Bioapatite,” Chemical Geology 451, no. 20 (2017): 183–188.

[18] Khoshakhlagh P, Rabiee SM, Kiaee G, , “Development and Characterization of a Bioglass/Chitosan Composite as an Injectable Bone Substitute,” Carbohydrate Polymers 157, no. 10 (2017): 1261–1271. 27987831 10.1016/j.carbpol.2016.11.003

[19] Liu S, Zhou H, Liu H, Ji H, Fei W, and Luo E, “Fluorine-Contained Hydroxyapatite Suppresses Bone Resorption Through Inhibiting Osteoclasts Differentiation and Function In Vitro and In Vivo,” Cell Proliferation 52, no. 3 (2019): e12613. 30968984 10.1111/cpr.12613 PMC6536412

[20] Pak CY, Zerwekh JE, and Antich P, “Anabolic Effects of Fluoride on Bone,” Trends in Endocrinology and Metabolism 6, no. 7 (1995): 229–234. 11540313 10.1016/1043-2760(95)00111-t

[21] Resmim CM, Dalpasquale M, Vielmo NI, , “Study of Physico-Chemical Properties and In Vitro Antimicrobial Activity of Hydroxyapatites Obtained From Bone Calcination,” Progress in Biomaterials 8, no. 1 (2019): 1–9. 30599070 10.1007/s40204-018-0105-2 PMC6425081

[22] Stábile FM, Albano MP, Garrido LB, Volzone C, De Oliveira PT, and Rosa AL, “Processing of ZrO2 Scaffolds Coated by Glass–Ceramic Derived From 45S5 Bioglass,” Ceramics International 42, no. 3 (2016): 4507–4516.

[23] Elliott JC, Structure and Chemistry of the Apatites and Other Calcium Orthophosphates, vol. 18 (Elsevier, 2013).

[24] Gross KA and Rodríguez-Lorenzo LM, “Sintered Hydroxyfluorapatites. Part II: Mechanical Properties of Solid Solutions Determined by Microindentation,” Biomaterials 25, no. 7–8 (2004): 1385–1394. 14643613 10.1016/s0142-9612(03)00636-7

[25] Demnati I, Grossin D, Marsan O, , “Comparison of Physical–Chemical and Mechanical Properties of Chlorapatite and Hydroxyapatite Plasma Sprayed Coatings,” Open Biomedical Engineering Journal 9 (2015): 42–55. 25893015 10.2174/1874120701509010042 PMC4391221

[26] Overgaard S, Bromose U, Lind M, Bünger C, and Søballe K, “The Influence of Crystallinity of the Hydroxyapatite Coating on the Fixation of Implants: Mechanical and Histomorphometric Results,” Journal of Bone & Joint Surgery 81, no. 4 (1999): 725–731. 10.1302/0301-620x.81b4.9282 10463753

[27] Joughehdoust S, Behnamghader A, Jahandideh R, and Manafi S, “Effect of Aging Temperature on Formation of Sol–Gel Derived Fluor-Hydroxyapatite Nanoparticles,” Journal of Nanoscience and Nanotechnology 10, no. 4 (2010): 2892–2896. 20355519 10.1166/jnn.2010.1397

[28] Laghzizil A, El Herch N, Bouhaouss A, Lorente G, and Macquete J, “Comparison of Electrical Properties Between Fluoroapatite and Hydroxyapatite Materials,” Journal of Solid State Chemistry 156, no. 1 (2001): 57–60.

[29] Pajor K, Pajchel L, and Kolmas J, “Hydroxyapatite and Fluorapatite in Conservative Dentistry and Oral Implantology—A Review,” Materials 12, no. 17 (2019): 2683. 31443429 10.3390/ma12172683 PMC6747619

[30] Gentleman E, Stevens MM, Hill R, and Brauer DS, “Surface Properties and Ion Release From Fluoride-Containing Bioactive Glasses Promote Osteoblast Differentiation and Mineralization In Vitro,” Acta Biomaterialia 9, no. 3 (2013): 5771–5779. 23128161 10.1016/j.actbio.2012.10.043 PMC5833947

[31] Marie PJ, De Vernejoul MC, and Lomri A, “Stimulation of Bone Formation in Osteoporosis Patients Treated With Fluoride Associated With Increased DNA Synthesis by Osteoblastic Cells In Vitro,” Journal of Bone and Mineral Research 7, no. 1 (1992): 103–113. 1549953 10.1002/jbmr.5650070115

[32] Jeyapalina S, Hillas E, Beck JP, Agarwal J, and Shea J, “Fluorapatite and Fluorohydroxyapatite Apatite Surfaces Drive Adipose-Derived Stem Cells to an Osteogenic Lineage,” Journal of the Mechanical Behavior of Biomedical Materials 125 (2022): 104950. 34740011 10.1016/j.jmbbm.2021.104950 PMC11822887

[33] Giannoudis PV, Dinopoulos H, and Tsiridis E, “Bone Substitutes: An Update,” Injury 36, no. 3 (2005): S20–S27. 16188545 10.1016/j.injury.2005.07.029

[34] Flierl MA, Smith WR, Mauffrey C, , “Outcomes and Complication Rates of Different Bone Grafting Modalities in Long Bone Fracture Nonunions: A Retrospective Cohort Study in 182 Patients,” Journal of Orthopaedic Surgery and Research 8, no. 1 (2013): 33. 24016227 10.1186/1749-799X-8-33 PMC3847297

[35] Ardisson A, de Senna PM, Granato R, Bergamo ET, Bonfante EA, and Marin C, “Success Rate of Mandible Implants Placed in Vascularized Fibula Bone Graft: A Systematic Review,” Journal of Oral Implantology 49, no. 1 (2023): 85–92. 35446964 10.1563/aaid-joi-D-20-00104

[36] Win KZ, Pimkhaokham A, and Kaboosaya B, “Comparing Bone Graft Success, Implant Survival Rate, and Marginal Bone Loss: A Retrospective Study on Materials and Influential Factors,” Journal of Oral Implantology 50, no. 4 (2024): 300–307. 38686547 10.1563/aaid-joi-D-23-00165

[37] Alhilou A, Do T, Mizban L, Clarkson BH, Wood DJ, and Katsikogianni MG, “Physicochemical and Antibacterial Characterization of a Novel Fluorapatite Coating,” ACS Omega 1, no. 2 (2016): 264–276. 27656690 10.1021/acsomega.6b00080 PMC5026462

[38] Marquis RE, “Antimicrobial Actions of Fluoride for Oral Bacteria,” Canadian Journal of Microbiology 41, no. 11 (1995): 955–964. 7497353 10.1139/m95-133

[39] Erlangga M, Charlena C, and Suparto IH, “Synthesis and Characterization of Fluorapatite-Copper(II) Oxide With Sol–Gel Method as an Antibacterial Biomaterial,” Jurnal Kimia Sains dan Aplikasi 27, no. 4 (2024): 8.

[40] Shanmugam S and Gopal B, “Copper Substituted Hydroxyapatite and Fluorapatite: Synthesis, Characterization and Antimicrobial Properties,” Ceramics International 40, no. Pt A (2014): 15655–15662.

[41] Ciobanu CS, Predoi D, Iconaru SL, , “Copper Doped Hydroxyapatite Nanocomposite Thin Films: Synthesis, Physico-Chemical and Biological Evaluation,” BioMetals 37, no. 6 (2024): 1487–1500. 39073689 10.1007/s10534-024-00620-2 PMC11618323

[42] Lysenko O, Dubok O, Borysenko A, and Shinkaruk O, “The Biological Properties of the Silver- and Copper-Doped Ceramic Biomaterial,” Journal of Nanoparticle Research 17, no. 4 (2015): 178.

[43] Mitra D, Kang E-T, and Neoh KG, “Antimicrobial Copper-Based Materials and Coatings: Potential Multifaceted Biomedical Applications,” ACS Applied Materials & Interfaces 12, no. 19 (2020): 21159–21182. 31880421 10.1021/acsami.9b17815

[44] Salah I, Parkin IP, and Allan E, “Copper as an Antimicrobial Agent: Recent Advances,” RSC Advances 11, no. 30 (2021): 18179–18186. 35480904 10.1039/d1ra02149d PMC9033467

[45] Vincent M, Duval RE, Hartemann P, and Engels-Deutsch M, “Contact Killing and Antimicrobial Properties of Copper,” Journal of Applied Microbiology 124, no. 5 (2018): 1032–1046. 29280540 10.1111/jam.13681

[46] Kazimierczak P, Wessely-Szponder J, Palka K, Barylyak A, Zinchenko V, and Przekora A, “Hydroxyapatite or Fluorapatite—Which Bioceramic Is Better as a Base for the Production of Bone Scaffold? A Comprehensive Comparative Study,” International Journal of Molecular Sciences 24, no. 6 (2023): 5576. 36982648 10.3390/ijms24065576 PMC10059826

[47] Nielson CR, Griffin A, Shea J, , “Fluorapatite Scaffolds With Gyroid Architecture: Advancing Synthetic Bioceramics for Bone Defect Repair in Cranioplasty,” Plastic and Reconstructive Surgery. Global Open 13, no. S1 (2025): 22–23.

[48] Taktak R, Elghazel A, Bouaziz J, Charfi S, and Keskes H, “Tricalcium Phosphate–Fluorapatite as Bone Tissue Engineering: Evaluation of Bioactivity and Biocompatibility,” Materials Science and Engineering: C 86 (2018): 121–128. 29525087 10.1016/j.msec.2017.11.011

[49] Bazin T, Magnaudeix A, Mayet R, , “Sintering and Biocompatibility of Copper-Doped Hydroxyapatite Bioceramics,” Ceramics International 47, no. 10 (2021): 13644–13654.

[50] Kamonwannasit S, Futalan CM, Khemthong P, Butburee T, Karaphun A, and Phatai P, “Synthesis of Copper-Silver Doped Hydroxyapatite via Ultrasonic Coupled Sol-Gel Techniques: Structural and Antibacterial Studies,” Journal of Sol-Gel Science and Technology 96, no. 2 (2020): 452–463.

[51] Benali Y, Predoi D, Rokosz K, , “Physico-Chemical Properties of Copper-Doped Hydroxyapatite Coatings Obtained by Vacuum Deposition Technique,” Materials 17, no. 15 (2024): 3681. 39124344 10.3390/ma17153681 PMC11313284

[52] Patil TV, Patel DK, and Lim KT, “In Vitro Osteogenic Response to Copper-Doped Eggshell-Derived Hyroxyapatite With Macrophage Supplements,” Journal of Biomedical Materials Research. Part A 113, no. 1 (2025): e37838. 39610338 10.1002/jbm.a.37838

[53] Tao B, Lin C, Guo A, , “Fabrication of Copper Ions-Substituted Hydroxyapatite/Polydopamine Nanocomposites With High Antibacterial and Angiogenesis Effects for Promoting Infected Wound Healing,” Journal of Industrial and Engineering Chemistry 104 (2021): 345–355.

[54] Yu W, Sun T-W, Ding Z, , “Copper-Doped Mesoporous Hydroxyapatite Microspheres Synthesized by a Microwave-Hydrothermal Method Using Creatine Phosphate as an Organic Phosphorus Source: Application in Drug Delivery and Enhanced Bone Regeneration,” Journal of Materials Chemistry B 5, no. 5 (2017): 1039–1052. 32263882 10.1039/c6tb02747d

[55] Ai F, Chen L, Yan J, , “Hydroxyapatite Scaffolds Containing Copper for Bone Tissue Engineering,” Journal of Sol-Gel Science and Technology 95, no. 1 (2020): 168–179.

[56] Elahi P, Steyl S, Shea J, Peter Beck J, Agarwal J, and Jeyapalina S, “Optimization of Antimicrobial Efficacy and Biocompatibility in Fluorapatite Bone Scaffolds Through Low-Dose Copper Doping for Advanced Bone Graft Applications,” Plastic and Reconstructive Surgery. Global Open 13, no. S1 (2025): 37.

[57] Milojkov DV, Radosavljević-Mihajlović AS, Stanić VD, , “Synthesis and Characterization of Luminescent Cu(2+)-Doped Fluorapatite Nanocrystals as Potential Broad-Spectrum Antimicrobial Agents,” Journal of Photochemistry and Photobiology. B 239 (2023): 112649. 10.1016/j.jphotobiol.2023.112649 36669353

[58] Brokesh AM and Gaharwar AK, “Inorganic Biomaterials for Regenerative Medicine,” ACS Applied Materials & Interfaces 12, no. 5 (2020): 5319–5344. 31989815 10.1021/acsami.9b17801

[59] Habibovic P and Barralet JE, “Bioinorganics and Biomaterials: Bone Repair,” Acta Biomaterialia 7, no. 8 (2011): 3013–3026. 21453799 10.1016/j.actbio.2011.03.027

[60] O'Neill E, Awale G, Daneshmandi L, Umerah O, and Lo KWH, “The Roles of Ions on Bone Regeneration,” Drug Discovery Today 23, no. 4 (2018): 879–890. 29407177 10.1016/j.drudis.2018.01.049

[61] Shen Q, Qi Y, Kong Y, , “Advances in Copper-Based Biomaterials With Antibacterial and Osteogenic Properties for Bone Tissue Engineering,” Frontiers in Bioengineering and Biotechnology 9 (2022): 795425. 35127670 10.3389/fbioe.2021.795425 PMC8811349

[62] Rondanelli M, Faliva MA, Infantino V, , “Copper as Dietary Supplement for Bone Metabolism: A Review,” Nutrients 13, no. 7 (2021): 2246. 34210051 10.3390/nu13072246 PMC8308383

[63] Sierpinska T, Konstantynowicz J, Orywal K, Golebiewska M, and Szmitkowski M, “Copper Deficit as a Potential Pathogenic Factor of Reduced Bone Mineral Density and Severe Tooth Wear,” Osteoporosis International 25, no. 2 (2014): 447–454. 23797848 10.1007/s00198-013-2410-x PMC3906556

[64] Uauy R, Olivares M, and Gonzalez M, “Essentiality of Copper in Humans,” American Journal of Clinical Nutrition 67, no. 5 (1998): 952S–959S. 9587135 10.1093/ajcn/67.5.952S

[65] Jeyapalina S, Steyl SK, Elahi P, , “Efficacy of Zinc-, Copper-, and Silver-Doped Fluorapatite as Bacteriophobic Surfaces for Percutaneous Osseointegrated Device Applications,” ChemNanoMat 12, no. 4 (2026): e202500276. 10.1002/cnma.202500276 PMC13479897 42609936

[66] Steyl SK, Jeyapalina S, Griffin A, , “Efficacy of Sintered Zinc-Doped Fluorapatite Scaffold as an Antimicrobial Regenerative Bone Filler for Dental Applications,” Journal of Dentistry 146 (2024): 105070. 38740251 10.1016/j.jdent.2024.105070 PMC11180563

[67] Elahi P, Winterholler E, Horsley J, and Sparks T, “The Influence of Sintering Condition on Microstructure, Phase Composition, and Electrochemical Performance of the Scandia-Ceria-Co-Doped Zirconia for SOFCs,” Science of Sintering 55 (2023): 237–258.

[68] Schneider CA, Rasband WS, and Eliceiri KW, “NIH Image to ImageJ: 25 Years of Image Analysis,” Nature Methods 9, no. 7 (2012): 671–675. 22930834 10.1038/nmeth.2089 PMC5554542

[69] Petri WH 3rd and Schaberg SJ, “The Effects of Antibiotic-Supplemented Bone Allografts on Contaminated, Partially Avulsive Fractures of the Canine Ulna,” Journal of Oral and Maxillofacial Surgery 42, no. 11 (1984): 699–704. 6436455 10.1016/0278-2391(84)90416-6

[70] Stanford R, Solomon M, Levick M, Kohan L, and Bell S, “Sterilization of Contaminated Bone-Tendon Autografts Using 10% Povidone–Iodine Solution,” Orthopedics 22, no. 6 (1999): 601–604. 10386802 10.3928/0147-7447-19990601-10

[71] Stepanovic Z and Ristic BM, “Bacterial Infections Associated With Allogenic Bone Transplantation,” Vojnosanitetski Pregled 72, no. 5 (2015): 427–430. 26165050 10.2298/vsp1505427s

[72] Horti N, Kamatagi M, Patil N, Sannaikar M, and Inamdar S, “Synthesis and Optical Properties of Copper Oxide Nanoparticles: Effect of Solvents,” Journal of Nanophotonics 14, no. 4 (2020): 046010.

[73] Wei M, Evans JH, Bostrom T, and Grøndahl L, “Synthesis and Characterization of Hydroxyapatite, Fluoride-Substituted Hydroxyapatite and Fluorapatite,” Journal of Materials Science. Materials in Medicine 14, no. 4 (2003): 311–320. 15348455 10.1023/a:1022975730730

[74] Shannon RD, “Revised Effective Ionic Radii and Systematic Studies of Interatomic Distances in Halides and Chalcogenides,” Acta Crystallogr. Sect. A 32, no. 5 (1976): 751–767.

[75] Guo C, Li L, Li S, Wang Y, and Yu X, “Preparation, Characterization, Bioactivity and Degradation Behavior In Vitro of Copper-Doped Calcium Polyphosphate as a Candidate Material for Bone Tissue Engineering,” RSC Advances 7, no. 67 (2017): 42614–42626.

[76] Noori A, Hoseinpour M, Kolivand S, , “Exploring the Various Effects of cu Doping in Hydroxyapatite Nanoparticle,” Scientific Reports 14, no. 1 (2024): 3421. 38341449 10.1038/s41598-024-53704-x PMC10858896

[77] Delgado-López JM, Frison R, Cervellino A, Gómez-Morales J, Guagliardi A, and Masciocchi N, “Crystal Size, Morphology, and Growth Mechanism in Bio-Inspired Apatite Nanocrystals,” Advanced Functional Materials 24, no. 8 (2014): 1090–1099.

[78] Bulina NV, Eremina NV, Vinokurova OB, Ishchenko AV, and Chaikina MV, “Diffusion of Copper Ions in the Lattice of Substituted Hydroxyapatite During Heat Treatment,” Materials 15, no. 16 (2022): 5759. 36013896 10.3390/ma15165759 PMC9415723

[79] Hossain MS, Tuntun SM, Bahadur NM, and Ahmed S, “Enhancement of Photocatalytic Efficacy by Exploiting Copper Doping in Nano-Hydroxyapatite for Degradation of Congo Red Dye,” RSC Advances 12, no. 52 (2022): 34080–34094. 36505682 10.1039/d2ra06294a PMC9704492

[80] Rodríguez-Lorenzo LM, Hart JN, and Gross KA, “Influence of Fluorine in the Synthesis of Apatites. Synthesis of Solid Solutions of Hydroxy-Fluorapatite,” Biomaterials 24, no. 21 (2003): 3777–3785. 12818550 10.1016/s0142-9612(03)00259-x

[81] Chaikina MV, Bulina NV, Prosanov IY, and Ishchenko AV, “Formation of Fluorapatite in the Equilibrium System CaO–P_2_O_5_–HF–H_2_O at 298 K in a Nitrogen Atmosphere,” Crystals 13, no. 8 (2023): 1264.

[82] Carlson GA, Dragoo JL, Samimi B, , “Bacteriostatic Properties of Biomatrices Against Common Orthopaedic Pathogens,” Biochemical and Biophysical Research Communications 321, no. 2 (2004): 472–478. 15358200 10.1016/j.bbrc.2004.06.165

[83] Jacobs A, Renaudin G, Charbonnel N, Nedelec J-M, Forestier C, and Descamps S, “Copper-Doped Biphasic Calcium Phosphate Powders: Dopant Release, Cytotoxicity and Antibacterial Properties,” Materials 14, no. 9 (2021): 2393. 34064435 10.3390/ma14092393 PMC8124198

[84] Weiss KM, Kucko SK, Mokhtari S, Keenan TJ, and Wren AW, “Investigating the Structure, Solubility, and Antibacterial Properties of Silver- and Copper-Doped Hydroxyapatite,” Journal of Biomedical Materials Research. Part B, Applied Biomaterials 111, no. 2 (2023): 295–313. 36054459 10.1002/jbm.b.35151

[85] Tuntun SM, Sahadat Hossain M, Uddin MN, Shaikh MAA, Bahadur NM, and Ahmed S, “Crystallographic Characterization and Application of Copper Doped Hydroxyapatite as a Biomaterial,” New Journal of Chemistry 47, no. 6 (2023): 2874–2885.

[86] Simon AT, Dutta D, Chattopadhyay A, and Ghosh SS, “Copper Nanocluster-Doped Luminescent Hydroxyapatite Nanoparticles for Antibacterial and Antibiofilm Applications,” ACS Omega 4, no. 3 (2019): 4697–4706. 31459656 10.1021/acsomega.8b03076 PMC6648608

[87] Hidalgo-Robatto BM, López-Álvarez M, Azevedo AS, , “Pulsed Laser Deposition of Copper and Zinc Doped Hydroxyapatite Coatings for Biomedical Applications,” Surface and Coatings Technology 333 (2018): 168–177.

[88] Bhattacharjee A, Fang Y, Hooper TJN, , “Crystal Chemistry and Antibacterial Properties of Cupriferous Hydroxyapatite,” Materials 12, no. 11 (2019): 1814. 31167438 10.3390/ma12111814 PMC6600772

[89] Mahmood Ul Hassan AH, Nooz N, Ashraf H, and Rehman OU, “Bacterial Pathogens in Orthopedic Implant Infection and Their Resistance to Antimicrobial Therapy: A Retrospective Analysis,” Journal of Orthopaedic Reports 4, no. 1 (2025): 100559.

[90] Morgenstern M, Erichsen C, Militz M, , “The AO Trauma CPP Bone Infection Registry: Epidemiology and Outcomes of *Staphylococcus aureus* Bone Infection,” Journal of Orthopaedic Research 39, no. 1 (2021): 136–146. 32720352 10.1002/jor.24804 PMC7749080

[91] Šístková J, Fialová T, Svoboda E, , “Insight Into Antibacterial Effect of Titanium Nanotubular Surfaces With Focus on *Staphylococcus aureus* and *Pseudomonas aeruginosa*,” Scientific Reports 14, no. 1 (2024): 17303. 39068252 10.1038/s41598-024-68266-1 PMC11283573

[92] Masters EA, Ricciardi BF, Bentley KLM, Moriarty TF, Schwarz EM, and Muthukrishnan G, “Skeletal Infections: Microbial Pathogenesis, Immunity and Clinical Management,” Nature Reviews Microbiology 20, no. 7 (2022): 385–400. 35169289 10.1038/s41579-022-00686-0 PMC8852989

[93] Prié H, Meyssonnier V, Kerroumi Y, , “*Pseudomonas aeruginosa* Prosthetic Joint-Infection Outcomes: Prospective, Observational Study on 43 Patients,” Frontiers in Medicine 9 (2022): 1039596. 36569155 10.3389/fmed.2022.1039596 PMC9774483

[94] Li Q, Song S, Li J, , “Antibacterial Properties and Biocompatibility of Hydroxyapatite Coating Doped With Various Cu Contents on Titanium,” Materials Transactions 63, no. 7 (2022): 1072–1079.

[95] Akbarpour MR, Farajnezhad F, Poureshagh AH, and Moniri JS, “Effects of Copper Doping on Fluorohydroxyapatite Coating: Analysis of Microstructure, Biocompatibility, Corrosion Resistance, and Cell Adhesion Characteristics,” Inorganic Chemistry 63, no. 43 (2024): 20314–20324. 39418538 10.1021/acs.inorgchem.4c01841

[96] Lu N, Zhang W, Weng Y, Chen X, Cheng Y, and Zhou P, “Fabrication of PDMS Surfaces With Micro Patterns and the Effect of Pattern Sizes on Bacteria Adhesion,” Food Control 68 (2016): 344–351.

[97] Hasan J and Chatterjee K, “Recent Advances in Engineering Topography Mediated Antibacterial Surfaces,” Nanoscale 7, no. 38 (2015): 15568–15575. 26372264 10.1039/c5nr04156b PMC4642214

[98] Zhu Y, The Surface Properties Effect on Bacterial Attachment and Biofilm Formation (Newcastle University, 2023).

[99] Carnicelli J, Effects of Surface Topography on Macrophages and Bacterial Cells (Syracuse University, 2022).

[100] Dasgupta S, Tarafder S, Bandyopadhyay A, and Bose S, “Effect of Grain Size on Mechanical, Surface and Biological Properties of Microwave Sintered Hydroxyapatite,” Materials Science & Engineering. C, Materials for Biological Applications 33, no. 5 (2013): 2846–2854. 23623105 10.1016/j.msec.2013.03.004

[101] Bennett BT, Beck JP, Papangkorn K, , “Characterization and Evaluation of Fluoridated Apatites for the Development of Infection-Free Percutaneous Devices,” Materials Science & Engineering. C, Materials for Biological Applications 100 (2019): 665–675. 30948103 10.1016/j.msec.2019.03.025

[102] Krawczynska AT, Michalicha A, Suchecki P, , “Enhancing Anti-Adhesion Properties by Designing Microstructure—The Microscopy and Spectroscopy Study of the Intercellular Bacterial Response,” Scientific Reports 14, no. 1 (2024): 24549. 39426980 10.1038/s41598-024-75045-5 PMC11490621

[103] Nikonam R, Sadrnezhaad SK, and Khaki JV, “Effect of Cu^2+^ Ion on Biological Performance of Nanostructured Fluorapatite Doped With Copper,” Scientia Iranica 24, no. 6 (2017): 2845–2855.

[104] Noori A, Hoseinpour M, Kolivand S, , “Exploring the Various Effects of cu Doping in Hydroxyapatite Nanoparticle,” Scientific Reports 14, no. 1 (2024): 1–13. 38341449 10.1038/s41598-024-53704-x PMC10858896

[105] Vo TTT, Peng T-Y, Nguyen TH, , “The Crosstalk Between Copper-Induced Oxidative Stress and Cuproptosis: A Novel Potential Anticancer Paradigm,” Cell Communication and Signaling 22, no. 1 (2024): 353. 38970072 10.1186/s12964-024-01726-3 PMC11225285

[106] Majhy B, Priyadarshini P, and Sen AK, “Effect of Surface Energy and Roughness on Cell Adhesion and Growth—Facile Surface Modification for Enhanced Cell Culture,” RSC Advances 11, no. 25 (2021): 15467–15476. 35424027 10.1039/d1ra02402g PMC8698786

[107] Hollinger JO and Kleinschmidt JC, “The Critical Size Defect as an Experimental Model to Test Bone Repair Materials,” Journal of Craniofacial Surgery 1, no. 1 (1990): 60–68. 1965154 10.1097/00001665-199001000-00011

[108] Schmelzeisen R, Boetel C, Schuberth HJ, and Pohlmeyer K, “Experimental Transplantation of Vascularized Autologous and Allogenic Bone Grafts for Mandibular Defects. Anatomical, Immunological and Surgical Basis for Vascularized Bone Transfer in the Gottingen Minipig,” International Journal of Oral and Maxillofacial Surgery 20, no. 4 (1991): 239–244. 1940503 10.1016/s0901-5027(05)80184-5

[109] Schon R, Schmelzeisen R, Shirota T, Ohno K, and Michi K, “Tissue Reaction Around Miniplates Used for the Fixation of Vascularized Iliac Crest Bone Grafts,” Oral Surgery, Oral Medicine, Oral Pathology, Oral Radiology, and Endodontology 83, no. 4 (1997): 433–440. 10.1016/s1079-2104(97)90141-4 9127373

[110] Williams DL, Haymond BS, Woodbury KL, , “Experimental Model of Biofilm Implant-Related Osteomyelitis to Test Combination Biomaterials Using Biofilms as Initial Inocula,” Journal of Biomedical Materials Research. Part A 100, no. 7 (2012): 1888–1900. 22492534 10.1002/jbm.a.34123 PMC3360822

